# Effect of orthopedic insoles on lower limb motion kinematics and kinetics in adults with flat foot: a systematic review

**DOI:** 10.3389/fbioe.2024.1435554

**Published:** 2024-07-09

**Authors:** Hairong Chen, Dong Sun, Yufei Fang, Shunxiang Gao, Qiaolin Zhang, István Bíró, Viktória Tafferner-Gulyás, Yaodong Gu

**Affiliations:** ^1^ Ningbo No. 2 Hospital, Ningbo, China; ^2^ Faculty of Sports Science, Ningbo University, Ningbo, China; ^3^ Doctoral School on Safety and Security Sciences, Óbuda University, Budapest, Hungary; ^4^ Faculty of Engineering, University of Szeged, Szeged, Hungary; ^5^ John von Neumann Faculty of Informatics, Óbuda University, Budapest, Hungary

**Keywords:** orthopedic insoles, flatfoot, adults, lower limb motion, kinematics, kinetics

## Abstract

Flatfoot is characterized by the collapse of the medial longitudinal arch, eversion of the rearfoot and abduction of the loaded forefoot. Orthopedic insoles are the frequently recommended treatment to support the arch of the foot, adjust the structure of the foot, reduce pain, improve stability and new techniques have been applied to the design of orthopedic insoles in recent years. However, the effectiveness of orthopedic insoles in different motions is still debated from the perspective of biomechanics. Therefore, this study aimed to explore the impact of orthopedic insoles on the kinematics and kinetics of lower limb motion, and to verify effectiveness and propose possible future research directions. We conducted a literature search across three databases employing Boolean operations and filtered results based on eligibility criteria. A total of 671 relevant literature were searched in this review, and 19 literature meeting the requirements were finally included. The results showed that: 1) orthopedic insoles were effective when patients walk, run and jump from the perspective of biomechanics; 2) orthopedic insoles had different result on the change of ankle sagittal angle, moment and peak pressure in the metatarsal region; 3) Whether the effect of insoles, which uses new techniques such as different 3D printed technologies and adds various accessories, can be further improved remains to be further studied; 4) Follow-up studies can pay more attention to the differences between diverse populations, increase the breadth of running and jumping and other movements research and long-term intervention.

## 1 Introduction

Flat foot, also known as pes planus, is a condition where the medial longitudinal arch of the foot collapses, leading to a flat appearance of the sole when standing (Flores et al., 2019). This condition can affect one or both feet and results in a series of biomechanical changes including the eversion of the rearfoot and the abduction of the loaded forefoot (Arachchige et al., 2019). Such alterations in foot structure can have significant repercussions for an individual’s overall posture and motor mechanics (Myerson, 1996; Van Boerum et al., 2003; Chinpeerasathian et al., 2024). Flat feet often lead to structural changes in the foot, causing various painful symptoms (Toullec et al., 2015). One common issue is plantar fascia pain, resulting from the additional stress placed on this thick band of tissue that spans the bottom of the foot, due to the collapsed arch (Chen, 2020). Similarly, the altered mechanics may exacerbate stress on the Achilles tendon, leading to Achilles tendonitis (Vijayakumar et al., 2017). The ligamentous structures of the foot may also become overstretched or unstable, particularly under the load during weight-bearing activities, contributing to overall joint laxity and instability (Clement et al., 1984; Bertani et al., 1999). The instability and pain in the foot often lead to rapid fatigability, limiting physical activity and endurance (Franco, 1987). Medial instability can contribute to an uneven distribution of weight, which not only aggravates foot discomfort but can also lead to pain in the knees, hips, and lower back. These musculoskeletal pains occur as the body attempts to compensate for the uneven weight distribution and altered biomechanics caused by flat foot (Messier et al., 1988; Kaufman et al., 1999; Herchenröder et al., 2021). For many adults experiencing these symptoms, orthopedic insoles are a frequently recommended treatment. These insoles are designed to support the foot’s arch, realign foot structure, alleviate pain, and enhance stability (Lusardi et al., 2012). The goal of using orthopedic insoles is to improve daily living and allow for greater physical activity without discomfort (Hume et al., 2008; Mills et al., 2010; Richter et al., 2011; Banwell et al., 2014).

At present, literature reviews have explored the effects of orthopedic insoles on the lower limbs. However, the impact of orthopedic insoles on flatfeet has been met with mixed conclusions in several systematic reviews (Michaudet et al., 2018; Choi et al., 2020). Assessing the effectiveness of orthotic devices is essential, considering their widespread prescription has considerable implications for healthcare expenses. For example, around 8% of the population, or five million people, were prescribed foot orthoses for medical reasons in Germany. This led to an increase of 466.6 million euros in statutory health insurance costs in 2019, attributable solely to these prescriptions (Herchenröder et al., 2021). Moreover, proof of either a beneficial or detrimental impact of orthoses on flatfeet can serve as a basis in orthopedic clinics to enhance the treatment strategies for patients suffering from flatfeet (Oerlemans et al., 2023).

Kinematic and radiological measurements have commonly been used as outcome measures in previous systematic reviews, with only a few focusing on kinetic parameters (Desmyttere et al., 2018; Choi et al., 2020; Hill et al., 2020). Moreover, most of the research concerns walking, whereas in daily activities people tend to wear orthopedic insoles for other movements such as running and jumping as well. There are few relevant reviews which focus on the effects of orthopedic insole on the kinematics and kinetics for different movements in population with flat feet.

Several new technological devices have been applied to the design and production of orthopedic insoles in the recent years, and biomechanical research on orthopedic insoles have also exploded. Against this background, my review systematically examines the biomechanical effects of orthopedic insoles on lower limb kinematics and kinetics in adults with flat feet, exploring their effectiveness and providing valuable references for future development and research directions in orthopedic insoles.

## 2 Materials and methods

This study was carried out in compliance with the PRISMA (systematic reviews and meta-analysis) preferred reporting items (Page et al., 2021; Zhou and Ugbolue, 2024; Feng and Song, 2017; Gao, 2022; Jiang, 2020).

### 2.1 Search strategy

To guarantee the reliability of the study findings, the search design was reviewed and endorsed by all the authors involved in the study. The electronic literature searches were conducted using the Web of Science, Scopus, and PubMed databases from 1 January 2019, to 26 March 2024. The selected time frame ensures that the study includes the most recent and relevant research, where newer studies can provide the latest insights and data. To enhance the comprehensiveness of our search, we employed a broad range of search terms and obtained a substantial number of results. We meticulously reviewed the retrieved literature to identify the studies that best met our criteria, ensuring the quality and validity of the included research. The search strategies were shown in Table 1.

**TABLE 1:** Search strategies in each electronic database.

Database	Search strategies	Result
PubMed	(“flat foot" [All Fields] OR “flatfoot" [All Fields] OR “flat feet" [All Fields] OR “flatfeet" [All Fields] OR “plane foot" [All Fields] OR “platypodia" [All Fields] OR “pes planus" [All Fields] OR “talipes planus" [All Fields]) AND (“inner sole" [All Fields] OR “insole" [All Fields] OR “orthopedic insole" [All Fields]) AND (“biomechanics" [All Fields] OR “kinetic" [All Fields] OR “kinematic" [All Fields]) AND 2019/01/01:2024/03/26 [Date - Publication]	12
Web of Science	TS= ((“flat foot” OR “flatfoot” OR “flat feet” OR “flatfeet” OR “plane foot” OR “platypodia” OR “pes planus” OR “talipes planus”) AND (“inner sole” OR “insole” OR “orthopedic insole”) AND (“biomechanics” OR “kinetic” OR “kinematic")) AND PY= (2019–2024)	20
Scopus	((“flat foot” OR “flatfoot” OR “flat feet” OR “flatfeet” OR “plane foot” OR “platypodia” OR “pes planus” OR “talipes planus”) AND (“inner sole” OR “insole” OR “orthopedic insole”) AND (“biomechanics” OR “kinetic” OR “kinematic")) AND PUBYEAR >2018 AND PUBYEAR <2025	639

### 2.2 Eligibility criteria

The inclusion criteria for screening the studies were as follows: 1) the articles must be published in English; 2) the article must appear in a peer-reviewed journal; 3) The participants must be adults (non-elderly and non-children); 4) the participants must suffer from flexible flatfoot; 5) the article must investigate lower limb motion kinematics and kinetics under insole intervention.

Articles were excluded if 1) articles were excluded if they were review articles; 2) Research subjects were excluded if they suffered from flexible flatfoot due to other diseases (e.g., rheumatoid arthritis, cerebral palsy); 3) articles were excluded if they focused solely on imaging research without addressing lower limb motion kinematics and kinetics; 4) articles were excluded if they focused on machine learning methods without direct examination of kinematics and kinetics; 5) articles were excluded if they only utilized finite element analysis or simulations without empirical data on human subjects.

### 2.3 Study risk of bias assessment

Two reviewers (H.C. and Y.G.) independently assessed the methodological quality of articles using the Combie criteria, which encompass seven domains (Kou et al., 2018). Based on the Combie evaluation tool, each article received a total possible score of 7.0 points, and quality of the articles was categorized as A, B, and C which indicated 6.0–7.0 points, 4.0–5.5 points, and 0–4.0 points, respectively. In the event of any disagreements during the quality assessment, an independent arbitrator would intervene to resolve them (I.B.).

### 2.4 Data extraction and management

Table 1 illustrates those two reviewers (H.C. and Y.G.) independently extracted data from all chosen studies using a standardized form according to the principles of participants, interventions, comparisons, and outcomes (PICOS).

## 3 Results

In this section, we presented the findings of our systematic review based on the Prisma framework, which provided a structured approach to categorize and analyze the results, ensuring a comprehensive and systematic examination of the data. The characteristic information of included studies were detailed in Table 2. Only studies that met the inclusion and exclusion criteria and had a risk of bias score of 5.5 or above were analyzed. We analyzed the impact of orthopedic insoles on lower limb walking, running, and jumping kinematics and kinetics in adults and aimed to present a clear and organized synthesis of the existing literature, highlighting key trends, gaps, and insights that emerged from our analysis.

**TABLE 2:** The characteristic information of included studies.

Author and year	Sample size (total)	Gender (Male/Female)	Experimental insole	Tests	Biomechanical parameters	Key findings
Han et al. (2019)	28	28/0	Normal insole (control) Type A (arch support function) Type B (arch support and cushion pads)	Walking speed: 80 beats per minute	Joint kinematics (Joint angle) Joint kinetics (Joint moment) GRF	Peak everted position (Type A, Type B↓VS Control) Range of rearfoot motion in the longitudinal axis (Type A↓VS Control) Peak evertor moment (Type A, Type B↓VS Control)
Ho et al. (2019)	26	26/0	Neutral flat insole Prefabricated foot orthose	Vertical countermovement jump (CMJ) Standing broad jump (SBJ)	Joint kinematics (Joint angle) Joint kinetics (Joint moment) GRF (Peak GRF)	CMJ take off: ankle eversion↓ SBJ take off: ankle eversion↓, peak horizontal GRF↓ peak ankle frontal moment↓
Karimi et al. (2019)	15	—	None Arch support foot insole	Walk with a comfortable speed	Joint kinematics Joint kinetics (Joint moment) GRF (Peak GRF)	First and second peaks of ankle adductor moment↓ Second peak of vertical force↓ First peak of anteroposterior↑ Mediolateral component↓
Lee et al. (2019)	12	—	None Customized arch support orthoses (CASO) Orthotic heel lift (HL)	Run on the runway at a self-selected speed	Joint kinematics (Joint angle) Joint kinetics (Joint moment, Achilles tendon force)	Peak dorsiflexion angle (CASO↑,HL↓VS None; CASO↑VS HL) Peak plantarflexion moment (CASO↓, HL↓VS None) Peak ATL (Achilles Tendon load) (CASO↓, HL↓VS None) Time to peak Achilles tendon force (CASO↑, HL↑VS None) Achilles tendon loading rate (CASO↓, HL↓VS None)
Lin et al. (2019)	12	4/8	Standard shoe (Shoe) Standard shoe with 3D Printed FOs (Shoe + FO)	Walk at a self-selected speed	Joint kinetics (Joint moment) GRF	Maximum ankle evertor moment↓ Peak external rotator moment↓ Maximum ankle plantar flexor moment↑
Xu et al. (2019)	90	—	3D printed customized (experimental group) Prefabricated insole (control group)	Walk at speed of 3.12 ± 1.95 km/h	Plantar pressure and kinetics (Peak pressure, Peak force)	Week 0: peak pressure in the 3rd metatarsal region↓ peak force in the 1st metatarsal↓,mid-foot pressure↑ Week 8: the peak pressure and force in mid-foot↑ Control group: the peak pressures of the 4th and 5th metatarsal areas↓, peak force of the 5th metatarsal area↓, peak force of 1st metatarsal area↑ (Week 8 VS Week 0) Experimental group: the peak pressure of the 2nd to 5th toe↑ the lateral heel area of the big toe↑ (Week 8 VS Week 0)
Zhai et al. (2019)	15	0/15	None Orthotic insole	Walk on level surface, walking up and down 10 cm and 20 cm stairs	Plantar pressure and kinetics (Peak force)	Plantar max force↓ (especially when walking downstairs)
Huang et al. (2020)	15	0/15	Arch-support insole Flat insole	Walk on treadmill: 9-degree incline (uphill walking) 9-degree decline (downhill walking), a level surface (level walking) at 2.7 km/h speed for all slopes	Plantar pressure and kinetics (Peak pressure)	Peak pressure of the BT↑ on the uphill and level surface Peak pressure of the MH↓ on the uphill, downhill, and level surface Peak pressure of the M2, M3 and M4↑ while the LH↓
Cheng et al. (2021)	10	4/6	Reinforced and undercut arch support (R + U+) Reinforced without undercut arch support (R + U-) Without reinforced and undercut arch support (R-U+) Control without insole	Walk at their comfortable speed	Joint kinematics (Joint angle) Plantar pressure and kinetics (Peak pressure, Pressure-time integral) GRF	Peak ankle dorsiflexion↑and peak pressure↑ at the medial midfoot region (insole vs. control) Peak pressure↓at the hindfoot region (insole vs. control) Peak medial midfoot pressure↑and medial hindfoot pressure↑(R + U- vs. R+U+ and R-U+) Peak pressure at lateral forefoot↓, lateral midfoot↑ (R+U+ vs. R + U- and R-U+) Pressure-time integral at the medial midfoot region (R-U+↑and R + U+↑vs. control, R + U+↓vs. R-U+)
Desmyttere et al. (2021)	19	6/13	Shoe only 3D printed flexible Fos +with posting 3D printed rigid Fos	Walk for 3 min at predetermined speed	Joint kinematics (Joint angle) Joint kinetics (Joint moment)	Midfoot eversion↓and forefoot abduction↓ (rigid foot orthoses vs. other) Ankle eversion angle↓, inversion moment↓and knee abduction moment↑(posting vs. other)
Jiang et al. (2021)	10	8/2	Ordinary flat insole General orthotic insole Plantar pressure redistribution insole (PPRI)	Walk on the treadmill at speed 0.8 m/s, 1.0 m/s, and 1.2 m/s	Plantar pressure and kinetics (Peak pressure)	Peak pressure in T area at slow speed (PPRI↑vs. flat insole) Peak pressure in M2 area at slow speed (PPRI↓vs. orthotic insole) Peak pressure in MH area at all speed (PPRI↓vs. flat insole) Peak pressure in MH area at slow speed (PPRI↓vs. orthotic insole) Peak pressure in MH area at fast speed (orthotic insole↓vs. flat insole) Peak pressure in LH area at normal speed (PPRI↓vs. flat insole) Peak pressure in LH area at all speed (orthotic insole↓vs. flat insole)
Khorasani et al. (2021)	18	8/10	Without insole Prefabricated soft insole Custom-molded rigid medical insole	Walk at a self-selected speed and rhythm	Plantar pressure and kinetics (Peak pressure, Peak force)	Mean pressure and force in the heel, MTP2,3 MTP4,5 (insole↓vs. control) Pressure in the medial midfoot (insole↑vs. control) No differences between two insoles
Ataabadi et al. (2022)	20	12/8	SHOD Custom-made FOs with medial heel wedge LDT (Low-dye tape)	Run at running speed (7.9 ± 0.6 km/h)	Joint kinematics (Joint angle) Joint kinetics (Joint moment)	Dorsiflexion angle (Fos↓vs. SHOD) Ankle eversion (Fos↓vs. SHOD and LDT) Knee adduction angle (Fos↑vs. SHOD and LDT) Hip adduction angle (LDT↑vs. Fos and SHOD) Hip external rotation (Fos↑vs. LDT) Plantar flexor moment (Fos↓vs. SHOD) Knee extensor moment (Fos↑vs. SHOD) Knee external rotation moment (Fos↑vs. SHOD and LDT) Knee abductor moment (Fos↑vs. SHOD) Hip extensor moment (LDT↑vs. Fos and SHOD)
Cherni et al. (2022)	19	6/13	Shoe only 3D printed flexible Fos 3D printed flexible FOs with posting 3D printed rigid Fos	Walk as normal as possible	Plantar pressure and kinetics (Peak pressure)	Peak pressures, mean pressures in the midfoot area (Fos↑vs. control) The latter was reinforced by increasing the stiffness
Ho et al. (2022)	12	5/8	Shoe without Fos Shoe with 3D printed Shoe with traditionally made Fos	Walk within 5% of their preferred time	Joint kinematics (Arch height drop) Joint kinetics (Joint moment, Joint power) GRF (Peak GRF)	Arch height drop (3D printed↓vs. traditional) Ankle plantarflexion moment (3D printed and traditional↓vs. control) Ankle power absorption (3D printed and traditional↓vs. control) VGRF (3D printed and traditional↓vs. control)
Hsu et al. (2022)	10	5/5	Without insoles Auto-scan insole Total contact insole Medial wedge insole	Walk at a self-selected speed	Joint kinematics (Navicular height, Joint angle) Joint kinetics	Navicular height↑, ankle dorsiflexion angle↑and comfort↑with insole Wedge insole was the most efficient in navicular height↑and ankle dorsiflexion angle↑
Jandova et al. (2022)	18	—	Original insole (INS1) Customized thermoplastic (INS2) 3D printed insole (INS3)	Walk	Plantar pressure and kinetics (Peak pressure)	Pressure in middle foot (INS1↓vs. INS2 and 3) Peak pressure in middle foot (35% higher in INS2 and 23% higher in INS3 compared to INS1) Peak pressure in the rearfoot (INS2↓vs. INS1)
Alsaafin et al. (2023)	31	0/31	Shoes only Foot orthoses (15° inverted angle) Foot orthoses (25° inverted angle)	Walk	Joint kinematics (Joint angle)	Maximum ankle plantarflexion angle maximum ankle dorsiflexion angle (foot orthose↓vs. shoe) Maximum ankle external rotation angle maximum ankle internal rotation angle (foot orthose↓vs. shoe) Maximum ankle plantarflexion angle (25°inverted angle↓vs. 15° inverted angle and shoe) Maximum hip external rotation angle (foot orthose↑ vs. shoe)
Dami et al. (2024)	15	—	Without Fos MWFOs (medially wedged Fos) TFOs (thin-flexible Fos)	Drop landing on the level surface Drop landing on the valgus inclined surface	Joint kinematics (Joint angle) Joint kinetics (Joint moment)	Level surface: midfoot dorsiflexion angle midfoot abduction angle (MWFOs↓vs. SHOD) Ankle eversion angle (MWFOs < TFOs < SHOD) Valgus inclined surface: midfoot dorsiflexion angle (MWFOs < TFOs < SHOD) Midfoot abduction angle (MWFOs↓ vs. TFOs and SHOD) Ankle eversion angle (MWFOs < TFOs < SHOD) Hip flexion angle (MWFOs↑vs. TFOs) Hip internal rotation angle (MWFOs↓vs. TFOs and SHOD) Hip abduction moment (MWFOs↓ and TFOs↓ vs. SHOD)

### 3.1 Literature selection

**FIGURE 1:**
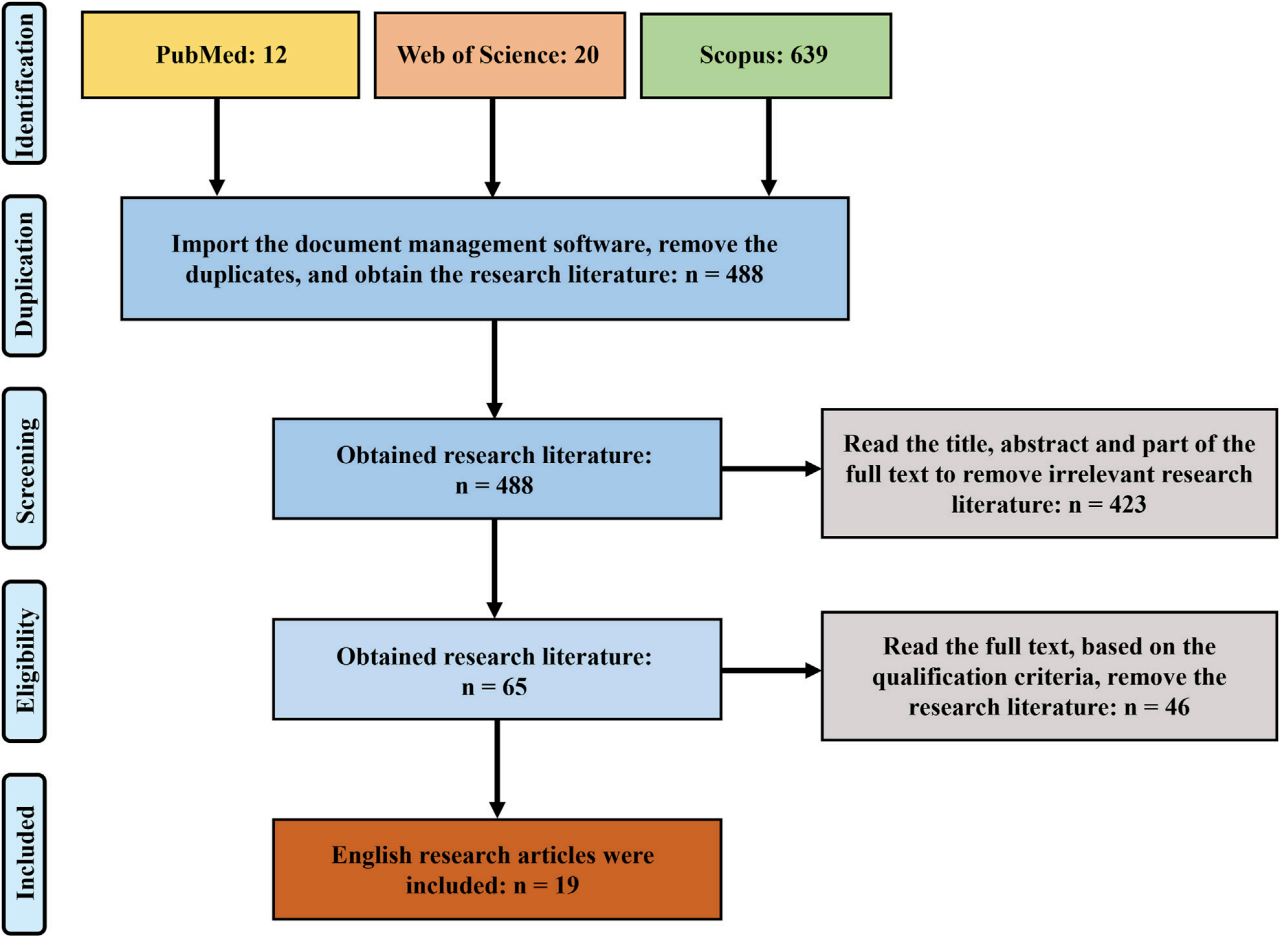
Prisma Flow Chart for Systematic review.

In the initial search, 671 articles were identified. After deduplication and pooling, 488 articles were advanced to the screening stage. Ultimately, 65 studies were chosen during the eligibility assessment, during which articles were excluded based on specific criteria if they suffered from flat feet due to other diseases (n = 2), imaging research (n = 18), children (n = 12), only used finite element analysis or simulations (n = 2), other foot orthotic interventions (n = 4), only used electromyogram analysis (n = 7), not lower limb motion kinematics and kinetics (n = 1), 19 articles were selected for the systematic review (Figure 1).

### 3.2 Original characteristics

**FIGURE 2:**
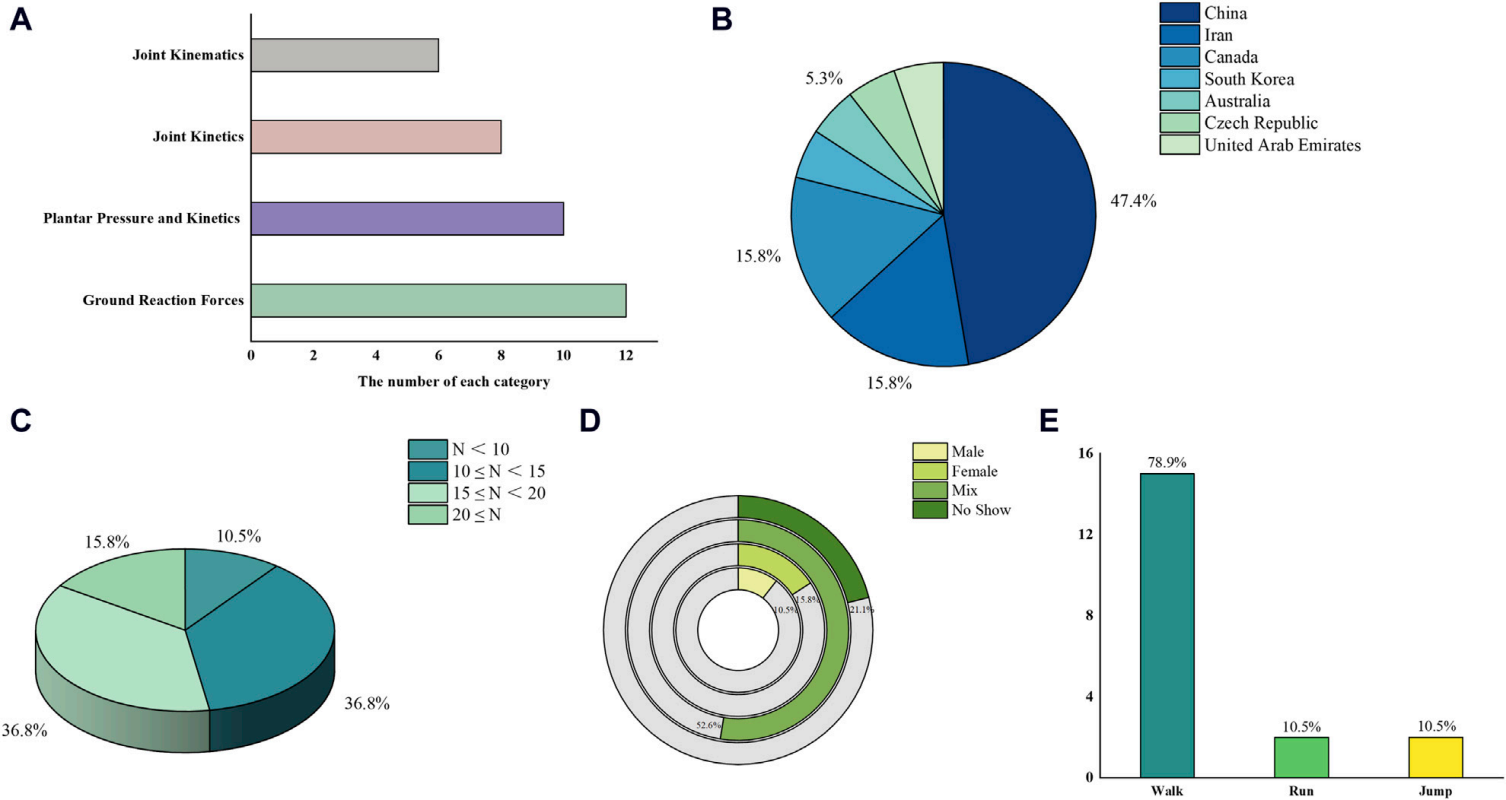
Characteristic information: **(A)** category number; **(B)** country of included studies; **(C)** sample size; **(D)** gender; **(E)** focused motion.

This review of studies on biomechanics reveals a focus on several key parameters: joint kinematics (12 studies), joint kinetics (10 studies), plantar pressure and kinetics (8 studies), and ground reaction forces (6 studies). Geographically, the distribution of these studies includes 47.4% from China, 15.8% from Iran and Canada, and 5.3% from South Korea, Australia, Czech Republic, and the United Arab Emirates. Sample sizes varied with 36.8% of studies including 15 to 20 participants, 36.8% including 10 to 15 participants, 10.5% including 5 to 10 participants, and 15.8% having more than 20 participants. In terms of participant gender, 10.5% of the studies exclusively involved males, 15.8% females, 52.6% a mix of both genders, and 21.1% did not describe. Most of the studies (78.9%) focused on walking, while 10.5% each focused on running and jumping (Figure 2).

### 3.3 Risk of bias

The risk of bias in all the selected studies was evaluated, and the outcomes are detailed in Table 3. Of the studies included, 94.7% scored above 5.5 points, which is categorized as quality A, while one study scored below 6.0 points, classified as quality B. Thus, the selected literature for this study is rated as high and moderate in quality.

**TABLE 3:** Study risk of bias assessment.

Studies	①	②	③	④	⑤	⑥	⑦	Grade	Quality
Han et al. (2019)	Yes	Yes	Yes	Yes	Yes	Yes	Yes	7	A
Ho et al. (2019)	Yes	Yes	Yes	Yes	Yes	No	Yes	6	A
Karimi et al. (2019)	Yes	Yes	Yes	Yes	Yes	No	Yes	6	A
Lee et al. (2019)	Yes	Yes	Yes	Yes	Yes	Yes	Yes	7	A
Lin et al. (2019)	Yes	Yes	Yes	Yes	Yes	No	Yes	6	A
Xu et al. (2019)	Yes	Yes	Yes	Yes	Yes	No	Yes	6	A
Zhai et al. (2019)	Yes	Yes	Yes	Yes	Yes	No	Yes	6	A
Huang et al. (2020)	Yes	Yes	Yes	Yes	Yes	Yes	Yes	7	A
Cheng et al. (2021)	Yes	Yes	Yes	Yes	Yes	No	Yes	6	A
Desmyttere et al. (2021)	Yes	Yes	Yes	Yes	Yes	No	Yes	6	A
Jiang et al. (2021)	Yes	Yes	Yes	Unclear	Yes	Yes	Yes	6.5	A
Khorasani et al. (2021)	Yes	Yes	Yes	Yes	Yes	No	Yes	6	A
Ataabadi et al. (2022)	Yes	Yes	Yes	Yes	Yes	Yes	Yes	7	A
Cherni et al. (2022)	Yes	Yes	Yes	Yes	Yes	No	Yes	6	A
Ho et al. (2022)	Yes	Yes	Yes	Yes	Yes	Yes	Yes	7	A
Hsu et al. (2022)	Yes	Yes	Yes	Unclear	Yes	No	Yes	5.5	B
Jandova et al. (2022)	Yes	Yes	Yes	Yes	Yes	No	Yes	6	A
Alsaafin et al. (2023)	Yes	Yes	Yes	Yes	Yes	Yes	Yes	7	A
Dami et al. (2024)	Yes	Yes	Yes	Yes	Yes	Yes	Yes	7	A

**Note:** ①The study design was scientific and rigorous; ②The data collection strategy is reasonable; ③The research reports sample response rates; ④The total representativeness of samples was favorable; ⑤The research purpose and method are reasonable; ⑥The power of the test was reported; ⑦The statistical method was correct.

### 3.4 Impact on walking

This part mainly covered the analysis of existing studies on the changes in joint kinematics, joint kinetics, plantar pressure and kinetics, and ground reaction forces when walking in orthopedic insoles conditions. A total of fifteen studies were analyzed.

#### 3.4.1 Joint kinematics

Eight studies investigated the effects of insole interventions on lower limb joint kinematics during walking. One study found that arch support insoles led to smaller peak everted position and reduced rearfoot motion compared to normal insoles (Han et al., 2019). Another study reported that orthotic insoles improved the arch index both before and after long-term intervention during walking on level surfaces and stairs (Zhai et al., 2019). In comparison to wearing shoes only, using 3D printed foot orthoses resulted in increased peak ankle dorsiflexion (Cheng et al., 2021). Additionally, rigid 3D printed foot orthoses reduced midfoot eversion and forefoot abduction, while 3D printed foot orthoses with posting decreased ankle eversion angle (Desmyttere et al., 2021). In terms of arch support, 3D printed orthoses showed a smaller arch height drop compared to traditional orthoses (Ho et al., 2022). Furthermore, insoles were found to increase navicular height, and 3D printed insoles specifically increased ankle dorsiflexion angle, with the wedge condition being most effective (Hsu et al., 2022). The maximum ankle dorsiflexion angle during mid-stance, the maximum ankle external and internal rotation angles, and the maximum ankle plantarflexion angle during loading response were all reduced under orthotic settings when compared to wearing shoes alone. Furthermore, when compared to the 15° inverted angle and shoes-alone situations, the maximal ankle plantarflexion angle at toe-off only reduced in the 25° inverted angle condition. In contrast, under orthotic settings, the maximal hip external rotation angle rose (Alsaafin et al., 2023). It is worth noting that one study by Karimi did not discover any appreciable variations in lower limb kinematics (Karimi et al., 2019).

#### 3.4.2 Joint kinetics

Six studies examined how insole interventions impact lower limb joint kinetics during walking (Han et al., 2019). Arch support insoles resulted in lower peak evertor moments compared to normal insoles (Karimi et al., 2019). Foot insoles decreased the first and second peaks of adductor moments on the ankle joint. 3D printed foot orthoses (FOs) reduced the maximum ankle evertor moment by an average of 35% and significantly decreased the peak external rotator moment by 16%. Additionally, the maximum ankle plantar flexor moment with 3D printed FOs was significantly higher than with regular shoes (Lin et al., 2019). FOs with posting reduced ankle inversion moment and increased knee abduction moment (Desmyttere et al., 2021). Traditional and 3D printed foot orthoses showed lower ankle plantar flexion moment during control conditions and higher power absorption compared to control and traditional conditions (Ho et al., 2022). The study of Hsu was the only one that did not find differences in lower limb kinetic (Hsu et al., 2022).

#### 3.4.3 Plantar pressure and kinetics

Eight articles reported the impact of insole interventions on the planter pressure during walking. The long-term intervention study of insoles showed following. Peak force in the first metatarsal region and peak pressure in the third metatarsal region were initially lower in the tailored (EVA) insoles group than in the standard insoles group. By week 8, though, the group using the tailored (EVA) insoles had more midfoot force and peak pressure. Conversely, by week 8, the first metatarsal area’s peak force was greater and the fourth and fifth metatarsal areas’ peak pressures and forces were lower in the traditional insoles group than they had been in the previous week (Xu et al., 2019). Regarding flatfoot, orthotic insole treatment effectively decreased plantar pressure, especially during downhill walking (Zhai et al., 2019). In arch support insoles, the peak pressure of the big toe (BT) was higher on uphill and level surfaces compared to flat insoles, while the peak pressure of the mid-heel (MH) was lower on all surfaces. Additionally, the peak pressure of the second (M2), third (M3), and fourth (M4) metatarsals was higher in arch-support insoles than flat insoles, while the lateral heel (LH) pressure was lower in arch-support insoles (Huang et al., 2020). In comparison to the control, there was a rise in peak pressure at the medial midfoot region and a fall in the rearfoot region for the insole conditions. Additionally, at the medial midfoot and hindfoot regions, R+U− demonstrated a considerably higher peak pressure than R+U+ and R−U+. In comparison to R−U+ and R + U−, R+U+ exhibited a notably reduced peak pressure at the forefoot and a higher one at the midfoot on the lateral side. Only the medial midfoot region showed significant variations in the pressure-time integral, and paired comparisons revealed that R−U+ and R+U+ were different from the control. Moreover, compared to R−U+, R+U+ showed a substantially reduced pressure-time integral at the medial forefoot region (Cheng et al., 2021).

At modest walking speeds, the peak pressure of PPRI insoles in the T region was higher than the flat insole. Peak pressure in the M2 region was lower when walking slowly with the PPRI insole than when using the orthotic insole. Peak pressure in the MH region was lower when walking at slow, normal, and rapid walking speeds with the PPRI insole than when walking at slow walking speeds with the orthotic insole. Furthermore, at a normal walking speed, the peak pressure in the LH area was lower while using the PPRI footbed than when using the flat insole. At normal, fast, and slow walking speeds, the peak pressure with the orthotic insole was lower than with the flat insole (Jiang et al., 2021). The mean pressure and force at the second, third, fourth, and fifth metatarsophalangeal (MTP2,3,4,5) joints as well as the heel decreased with the usage of insoles. Nevertheless, it was discovered that wearing insoles increased pressure in the medial midfoot. There were no discernible variations in plantar pressure and force between the rigid insole that was custom-molded and the prefabricated soft insole (Khorasani et al., 2021). Walking with foot orthoses caused the greatest alterations in the midfoot area, where peak pressures, mean pressures, and contact area all increased (Cherni et al., 2022). In terms of pressure on the middle foot, both INS2 and INS3 result in increased peak pressure when compared to INS1, with INS2 having a 35% higher peak pressure and INS3 showing a 23% higher peak pressure in the middle foot region. For the rearfoot pressure, INS2 decreased the peak pressure when compared to the INS1(Jandova et al., 2022).

#### 3.4.4 Ground reaction force

Five articles investigated the effects of insole interventions on ground reaction forces during walking. One study discovered that using arch support insoles while walking led to a reduction in the second peak of vertical force, but an increase in the first peak of anteroposterior force. In addition, the sideways component of the force exerted by the ground decreased when participants utilized insoles, as opposed to walking without them (Karimi et al., 2019). Another study reported lower vertical ground reaction forces during walking with both Traditional and 3D printed insoles compared to a control condition (Ho et al., 2022). The studies of Han, Lin and Cheng did not find differences in ground reaction force (Han et al., 2019; Lin et al., 2019; Cheng et al., 2021).

### 3.5 Impact on running

This part mainly covered the analysis of existing studies on the changes in joint kinematics and joint kinetics when running in orthopedic insoles conditions. A total of two studies were analyzed.

#### 3.5.1 Joint kinematics

Two articles investigated the effects of insole interventions on lower limb joint kinematics during running. In the first study, he use of Heel Lifts (HL) caused the peak dorsiflexion angle to drop in comparison to the control condition, whereas the use of Custom Arch Support Orthotics (CASO) increased it. Furthermore, when employing CASO, the peak dorsiflexion angle was greater than when using HL (Lee et al., 2019). In the second study, when compared to wearing standard shoes (SHOD), foot orthotics (FOs) decreased the dorsiflexion angle and decreased ankle eversion during the whole stance phase. During the stance phase, FOs increased the knee adduction angle at the knee joint. Furthermore, in comparison to the Low-Dye Taping (LDT) condition, FOs enhanced hip external rotation in the horizontal plane at the start of the stance phase (Ataabadi et al., 2022).

#### 3.5.2 Joint kinetics

Two articles reported the impact of insole interventions on the kinetic of lower limb joints during running. The peak plantarflexion moment was reduced when running with both CASO and HL compared to the control condition. Additionally, peak Achilles tendon loading (ATL) was lower when using HL during running compared to the control. In comparison to the control, both the CASO and HL interventions demonstrated a marginally longer duration from initial contact to peak Achilles tendon force (duration to peak Achilles tendon force). Running with HL resulted in a lower Achilles tendon loading rate (ATLR) compared to the control group (Lee et al., 2019). The plantar flexor moment was lower with FOs compared to SHOD. Knee moments (extensor, external rotation, and abductor) were all higher with Fos. Additionally, the knee external rotation moment remained higher with FOs when compared to LDT. In contrast, the hip extensor moment was higher with LDT than with FOs and SHOD (Ataabadi et al., 2022).

### 3.6 Impact on jumping

This part mainly covered the analysis of existing studies on the changes in joint kinematics, joint kinetics and ground reaction forces when jumping in orthopedic insoles conditions. A total of two studies were analyzed.

#### 3.6.1 Joint kinematics

The impact of insole interventions on lower limb joint kinematics during jumping was examined in two articles. One study found that foot orthoses resulted in less ankle eversion during the take-off phase of a countermovement jump (CMJ) compared to an insole condition. Another study reported that during a standing broad jump (SBJ), foot orthoses were associated with less ankle eversion compared to the insole condition (Ho et al., 2019). Modified wedge foot orthoses (MWFOs) were found to reduce the midfoot dorsiflexion angle during the landing phase of drop landings on a level surface when compared to shoe wear. When using MWFOs as opposed to shoes, a reduced midfoot abduction angle was also observed. Furthermore, MWFOs and total contact foot orthoses (TFOs) both led to a smaller ankle eversion angle compared to shoes, with MWFOs also showing a smaller ankle eversion angle compared to TFOs. During drop landings on a valgus-inclined surface, both MWFOs and TFOs were associated with a smaller midfoot dorsiflexion angle compared to wearing shoes. Additionally, MWFOs showed a smaller midfoot dorsiflexion angle compared to TFOs. Moreover, MWFOs resulted in a smaller midfoot abduction angle compared to TFOs and shoes. Both MWFOs and TFOs were associated with a smaller ankle eversion angle compared to shoes, with MWFOs also showing a smaller ankle eversion angle compared to TFOs. Compared to TFOs, MWFOs showed a larger hip flexion angle, and compared to TFOs and shoes, MWFOs showed a smaller hip internal rotation angle (Dami et al., 2024).

#### 3.6.2 Joint kinetics

Two articles reported the impact of insole interventions on the kinetic of lower limb joints during jumping. Foot orthosis conditions was less peak ankle frontal moment than insole condition at standing broad jump (SBJ) (Ho et al., 2019). Dropping onto a level surface: A smaller ankle inversion moment was observed for individuals wearing minimal footwear (MWFOs) compared to those wearing shoes (shod). A smaller hip abduction moment was observed for individuals wearing toe-only footwear (TFOs) compared to MWFOs. Dropping onto a valgus inclined surface: A smaller hip abduction moment was observed for both TFOs and MWFOs compared to those wearing shoes (Dami et al., 2024).

#### 3.6.3 Ground reaction force

One article reported the impact of insole interventions on GRF during jumping. Foot orthosis conditions was lower peak horizontal GRF than insole condition at standing broad jump (SBJ) (Ho et al., 2019).

## 4 Discussion

In recent years, orthopedic insoles have received a lot of attention in the studies of lower limb biomechanics in motion. This systematic review categorized and sorted out the key information and findings of the studies, analyzed the effect of orthopedic insoles on lower limb motion kinematics and kinetics in adults with flatfoot including walking, running, and jumping movements, and aimed to reveal the effectiveness of orthopedic insoles in different motion forms and to provide a scientifically rigorous basis for the use of orthopedic insoles. In addition, the shortcomings of the existing studies were found to provide valuable directions for the future development and research direction of orthopedic insoles.

### 4.1 Characteristic information

From the perspective of the original characteristics, the countries of origin of selected articles were mainly concentrated in Asia and the sample size of the participants was mostly between 10 and 20. In addition, for the gender selection of the participants, most of the studies were mixed gender, but there are anatomical characteristics differences between males and females such as the pelvis, which may have an impact on the effect produced by orthopedic insoles (Petrie et al., 2023). In terms of the type of orthopedic insoles, the main types of insoles were common customized insoles, 3D printed insoles of various designs, and various accessories added for assistance. In addition, the main motion studied was walking, with relatively few studies on running and jumping motions.

### 4.2 Walking

#### 4.2.1 Kinematics

Almost overwhelmingly, studies had found that orthopedic insoles have an impact on walking kinematics. During walking, orthopedic insoles provided better arch support compared to no insoles and have a significant effect when walking up and down stairs (Ataabadi et al., 2022; Alsaafin et al., 2023). Orthopedic insoles reduced peak ankle eversion angle, maximum ankle external rotation angle, internal rotation angle, and increased maximum hip external rotation angle (Han et al., 2019; Jandova et al., 2022). These kinematic changes, which help to reduce the abnormal posture of flatfoot, help flatfoot patients to perform walking. However, the effects produced by different designs of orthopedic insoles on walking kinematics were controversial. Some studies had found that the use of 3D printed orthopedic insoles can reduce the arch height drop better compared to traditional orthopedic insoles, but the effect of different designs of 3D printed insoles on the reduction of the arch height drop was not consistent. The wedge insoles had the best effect, and the effect of the other designs of 3D printed insoles had no difference (Khorasani et al., 2021; Ataabadi et al., 2022). The effects of different designs of 3D printed insoles on reducing peak ankle eversion angle were also inconsistent. The 3D printed orthopedic insoles with posting was the most effective, but there was no difference in effect between the other designs of 3D printed insoles. The rigid 3D printed orthopedic insoles, compared to no insoles and other types of 3D printed insoles reduced midfoot eversion and forefoot abduction (Lee et al., 2019). These suggested that changing the design of the insole may increase its alteration of walking kinematics and better correction of abnormal foot posture.

However, there were also opposing trends in the effects of different designs of orthopedic insoles on walking kinematics. Orthopedic insoles reduced the range of motion in the sagittal plane of the rearfoot, maximum ankle plantarflexion and dorsiflexion angle, while angle orthopedic insoles with a 25° inverted further reduced the maximum ankle plantarflexion angle (Han et al., 2019; Jandova et al., 2022). However, 3D printed insoles increased peak ankle dorsiflexion angle and the effect of different designs of 3D printed insoles on increasing the peak ankle dorsiflexion angle was not consistent, with wedge insole increasing the most significantly but there was no disparity in the effect between other designs of 3D printed insoles (Ho et al., 2019; Ataabadi et al., 2022). There was still controversy about how ankle angles change to benefit population with flatfoot. One view was that flatfoot was due to excessive dorsiflexion and eversion resulting in talus and tarsal subluxation and pronation, where dorsiflexion or hypermobility of the ankle should be controlled (Needleman and international, 2005). Another view was that the limited range of motion of the ankle leads to rearfoot inversion and that the range of motion of the ankle should be restored (Hösl et al., 2014). Therefore, it remained to be explored which design of orthopedic insoles was more effective in modifying ankle dorsiflexion and extension.

#### 4.2.2 Kinetics

The vast proportion of studies had found that orthopedic insoles influenced walking joint kinetics. Orthopedic insoles exhibited smaller peak ankle eversion moment and ankle power absorption; 3D printed insoles similarly exhibited reduced maximal ankle eversion moment, peak external rotator moment, and ankle power absorption (Han et al., 2019; Khorasani et al., 2021; Ho et al., 2022). The magnitude of joint moments during walking can be considered key to preventing injury, and using fewer moments will reduce the likelihood of injury from overuse (Novacheck and posture, 1998). Increasing the stiffness of 3D printed Fos will reduce the ankle inversion moment and the functional demands on the inversion muscles will be reduced, which may result in overuse injuries to other compensatory muscles. However, a rise in the knee abduction moment, despite values within the safe range, may have an undesirable effect and lead to the development of osteoarthritis in the medial compartment knee (Lee et al., 2019). Like joint kinematics, opposing results existed in studies regarding ankle sagittal plane moment. 3D printed insole showed greater ankle plantarflexion moment, whereas traditionally made insoles and 3D printed insoles showed smaller ankle plantarflexion moment (Khorasani et al., 2021; Ho et al., 2022). The divergence of the studies lies in the understanding of muscle activation of the peroneus longus, peroneus brevis, soleus, medial peroneus, and lateral peroneus in patients with flatfoot during the stance phase, which can be further validated by electromyogram in subsequent studies.

#### 4.2.3 Plantar pressure and kinetics

Orthopedic insoles affected plantar pressure and kinetics during walking. The impact of orthopedic insoles on plantar pressure and kinetics typically included a rise in peak pressure at the midfoot and a reduction in peak pressure at the heel (Ho et al., 2019; Xu et al., 2019; Jiang et al., 2021). Orthopedic insoles helped maintain the calcaneus in a normalized alignment, improving pressure distribution and absorption. With the addition of longitudinal arch support, these insoles shifted pressure from the rearfoot to the midfoot and decreased rearfoot pressure, ultimately reducing the risk of tibial stress fractures (Aminian et al., 2013). The intervention of orthopedic insoles can normalize plantar pressure distribution. Different designs of orthopedic insoles caused further effects on plantar pressure distribution. Customized insoles and 3D printed insoles resulted in a further increase in midfoot peak pressure, a decrease in rearfoot peak pressure, and a further increase in midfoot peak pressure after long term intervention (Ho et al., 2019; Cherni et al., 2022; Hsu et al., 2022). Peak pressure in the metatarsal region is still a controversial topic. According to recent research, using a medial longitudinal arch support may help to transfer pressure from the first metatarsal to the second and third metatarsals in the central forefoot. Additionally, this support may lessen forefoot pronation and lessen strain on the first metatarsal by keeping the subtalar joint in a position that is similar to its usual state (Redmond et al., 2009). After long term intervention, 3D printed insoles reduced peak pressure and force in the medial metatarsal region (Hsu et al., 2022). However, it had been shown that orthopedic insoles had no impact on pressure and force distribution in the M1 region (Ho et al., 2019; Xu et al., 2019; Jiang et al., 2021). Subsequent studies should pay more attention to this point. Different walking styles and different walking speeds were also factors that influenced the alteration of plantar pressure by orthopedic insoles. Pressure distribution in the rearfoot and metatarsal region was altered as a result (Karimi et al., 2019; Lin et al., 2019; Alsaafin et al., 2023).

#### 4.2.4 Kinematics

There were not many studies related to the effect of orthopedic insoles on ground reaction forces during walking and most of them reported no difference existed (Han et al., 2019; Ho et al., 2019; Ho et al., 2022). Studies showed that aligning the foot structure with orthopedic insoles resulted in a decrease in the peak vertical GRF (Cheng et al., 2021; Khorasani et al., 2021). Further studies should follow to determine the effects.

### 4.3 Running

Orthopedic insoles had been shown to correct abnormal foot plantarflexion moment, decrease achillea tendon forces, and redress abnormal ankle eversion foot posture during running, but adding the medial heel wedge results in increased knee moment. Like walking, what effect orthopedic insoles have on the peak dorsiflexion angle still need to be further investigated, and different designs of orthopedic insoles affected the peak dorsiflexion angle differently (Huang et al., 2020; Desmyttere et al., 2021).

### 4.4 Jumping

In jumping and landing motions, orthopedic insoles can reduce foot and ankle eversion and increase foot stability. In addition, orthopedic insoles reduced ankle stress and enhance hip stability in landing motion. Different designs of orthopedic insoles have different effects on hip joint biomechanics (Zhai et al., 2019; Dami et al., 2024).

### 4.5 Limitations and directions

The review primarily focused on walking, running, and jumping, while overlooking other critical activities such as stair climbing, lateral movements, or sports-specific actions. Methodological variations among included studies, including differences in orthopedic insole types, could impact result comparability. The review may not adequately address outcome variations due to small sample sizes or limited diversity in study populations, including differences in age, weight, and activity levels. Most studies emphasized short-term effects of orthopedic insole usage, neglecting long-term durability and sustained biomechanical impacts.

Future research should encompass a broader range of movements and activities to offer the more comprehensive assessment of effectiveness. Standardized protocols for biomechanical analysis and insole design would enhance cross-study comparisons. Long-term studies are essential to evaluate sustained effects on lower limb biomechanics and foot health. Studies should also encompass larger, more diverse populations to explore how factors such as age, gender, weight, and activity levels influence orthopedic insole efficacy. Lastly, investigating and comparing new technologies and materials in orthopedic insole design would advance understanding in this field.

Some specific examples of potential studies or experiments that could further advance. Recruit a diverse cohort that encompasses a wide range of ages, genders, weights, and physical activity levels, ensuring a robust sample size to boost the study’s statistical strength. Implement a variety of orthopedic insole types, including traditional materials as well as innovative technologies like 3D printing and specialized accessories. Conduct longitudinal studies with follow-up periods from 6 months to 2 years to evaluate both the immediate and prolonged impacts of the insoles. Gather extensive kinematic and kinetic data during various activities, including walking, running, jumping, stair climbing, and lateral movements, utilizing standardized biomechanical assessment tools and protocols. Apply sophisticated statistical methods to dissect the effects of orthopedic insoles on lower limb biomechanics and perform subgroup analyses to identify differences based on gender, age, and other demographic variables. These strategic methodologies will enable future research to deliver more conclusive and relevant findings on the efficacy of orthopedic insoles for patients with flatfoot.

### 4.6 Summary

Overall, recent studies have demonstrated the effectiveness of orthopedic insoles during walking, running, jumping motions, correcting abnormal foot postures, and better protecting patients for these motions. However, there was still disagreement about changes in peak ankle eversion angle, ankle sagittal plane moment, and peak pressure in the metatarsal region. No consensus on whether the biomechanical effectiveness of the insole can be further enhanced using personalization, 3D printed, and the addition of a variety of accessories, which could be further explored in subsequent studies to find the most effective orthopedic insoles. In addition, follow up studies could focus more on diverse populations, jumping and other motions and long-term intervention, etc.

## 5 Conclusion

Based on the results of this systematic review we can conclude the effectiveness of orthopedic insoles for walking, running, and jumping. However, the changes of orthopedic insoles on the sagittal plane angle and moment of ankle joint and the peak pressure in the metatarsal region are still controversial. It is unknown whether the orthopedic insoles designed by the new technology can improve the effect. Besides, it is necessary to expand the depth of studies related to the more diverse populations, the running, jumping and other motions and long-term intervention, etc. These are the directions for the development and research of orthopedic insoles in the future.
